# Human CAFs promote lymphangiogenesis in ovarian cancer via the Hh-VEGF-C signaling axis

**DOI:** 10.18632/oncotarget.18621

**Published:** 2017-06-27

**Authors:** Rui Wei, Mengqin Lv, Fei Li, Teng Cheng, Zhengzhong Zhang, Guiying Jiang, Ying Zhou, Ruiqiu Gao, Xiao Wei, Jicheng Lou, Xizi Wu, Danfeng Luo, Xiangyi Ma, Jin Jiang, Ding Ma, Ling Xi

**Affiliations:** ^1^ Cancer Biology Research Center, Key Laboratory of The Ministry of Education, Tongji Hospital, Tongji Medical College, Huazhong University of Science and Technology, Wuhan, People's Republic of China; ^2^ Department of Molecular Biology, UT Southwestern Medical Center at Dallas, TX, Dallas, USA

**Keywords:** ovarian cancer, hedgehog signaling, cancer associated fibroblasts, VEGF-C, lymphangiogenesis

## Abstract

Cancer-associated fibroblasts (CAFs) play a pivotal role in the development and progression of many human cancers. Recent studies have shown that Hedgehog (Hh) signalling modulates the stromal microenvironment and prepares a suitable niche for tumour metastasis. However, the detailed molecular mechanisms underlying CAF-mediated lymphangiogenesis have not been fully elucidated. Therefore, our goal is to illustrate whether Hh ligands can activate Hh signalling in CAFs in a paracrine fashion and elucidate the effect of CAFs on lymphangiogenesis. We determined here that Sonic Hedgehog (SHH) secreted by ovarian cancer (OC) cells activated Hh signalling in CAFs and promoted the proliferation of CAFs. Moreover, we co-injected SHH-overexpressing OC cells and CAFs in a xenograft model and found that the CAFs accelerated tumourigenesis and lymphangiogenesis in OC. Mechanistically, we found that SHH secreted by the OC cells induced VEGF-C expression in CAFs. Inhibition of Hh signalling in CAFs decreased VEGF-C expression and diminished the positive role of CAFs in supporting tumourigenesis and lymphangiogenesis in a murine xenograft model. Our results demonstrate that CAFs constitute a supportive niche for cancer lymphangiogenesis via the Hh/VEGF-C signalling axis and provide evidence for the clinical application of Hh inhibitors in the treatment of OC.

## INTRODUCTION

Ovarian cancer (OC) is the most lethal gynaecologic malignancy with high mortality [1]. Each year, nearly 225,000 women are diagnosed with OC, and more than 140,000 patients die of OC worldwide. Although great progress has been made in the treatment of OC, recurrence of advanced OC is inevitable [2]. Among the advanced OC patients, especially those with lymph node metastasis, more than 70% relapse after 2 years of withdrawal of chemotherapy [3]. It is urgent to fully understand the mechanism of OC metastasis and find a new and efficient targeted therapy.

Cancer-associated fibroblasts (CAFs) originating from stromal fibroblastic cells are the dominating component of the tumour microenvironment [4]. CAFs have been shown to play a crucial role in tumour metastasis [5, 6]. Generally, the stroma interacts with tumour cells via reciprocal communications mediated by secretory factors. CAFs produce lactate, ketones, glutamine, fatty acids and cysteine to promote tumour formation and development [7, 8]. Thus, it is important to elucidate the molecular mechanisms underlying the function of CAFs in tumourigenesis. Recent data demonstrated the critical role of CAFs in the proliferation and metastasis of OC cells [9, 10]. Moreover, CAFs have been reported to promote tumour angiogenesis [11]. However, there is a limited number of studies examining CAFs in the lymphangiogenesis of OC, and the underlying mechanism involved remains obscure.

Tumour cells establish an autocrine–paracrine communication circuit with the stromal microenvironment through reciprocal signalling. Hedgehog (Hh) signalling was reported to be a potential signalling pathway in CAFs induced by tumour cells [12]. Previous reports suggested that Hh signalling effects tumourigenesis of various human cancers by controlling cell proliferation, angiogenesis and epithelial-mesenchymal transition (EMT) in an autocrine-juxtacrine manner [13, 14]. However, recent publications have described an alternative mechanism by which Hh ligands secreted by tumour cells activate Hh signalling in stromal cells in a paracrine manner rather than by stimulating the epithelial cells of the tumour [15]. These results highlight the importance of Hh signalling in the tumour microenvironment. For OC, few studies have focused on the paracrine effects of stromal Hh signalling on metastasis. Given the high incidence of lymph node metastasis in clinically advanced OC [16], we attempted to elucidate the connection between CAFs and Hh signalling in OC lymphangiogenesis.

In this study, we investigated the possible role of CAFs in promoting lymphangiogenesis via Hh signalling. We demonstrated that OC cell-derived Sonic Hedgehog (SHH) activated Hh signalling in CAFs, which increased the expression of VEGF-C and promoted the capillary tube formation of lymphatic endothelial cells (LECs) *in vitro*, and induced tumour lymphangiogenesis *in vivo*. Suppression of Hh signalling activity in CAFs attenuated the pro-lymphangiogenesis effects of CAFs. Our results demonstrate a role of Hh signalling in the tumour stroma as a regulator of lymphangiogenesis and highlight the important interplay between CAFs and endothelial cells. Thus, this study provides potential targets for the future development of novel therapeutics for OC.

## RESULTS

### SHH ligands can activate the Hh signalling of CAFs

**Figure 1:**
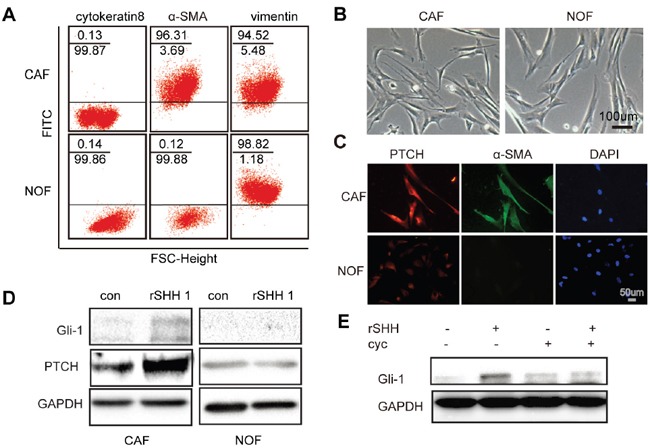
Exogenous SHH ligands can active Hh signalling in CAFs **(A)** Flow cytometric analysis of marker proteins in CAFs and NOFs demonstrating that CAFs are positive for α-SMA and vimentin but negative for cytokeratin 8, whereas NOFs are positive for vimentin but negative for α-SMA and cytokeratin 8. **(B)** Representative images of morphology of passage 3 CAFs and NOFs. **(C)** Representative fluorescent images of PTCH- (red), α-SMA- (green) and DAPI-labelled (blue) CAFs and NOFs. **(D)** Western blot analysis of Gli-1 and PTCH in CAFs and NOFs after 48 h treatment with rSHH (1 μg/ml). GAPDH served as the loading control. **(E)** Western blot analysis of Gli-1 and PTCH in CAFs in the absence or presence of rSHH (1 μg/ml) or Cyclopamine (10 μM) for 48 h.

Western blot analysis (WB) and immunohistochemistry (IHC) revealed that SHH levels were equally high in four different types of OC tissues, whereas the normal ovarian tissues hardly expressed SHH (Supplementary Figure 1A, 1B and 1C), suggesting that the expression of Hh ligand was aberrantly activated in epithelial OC and that CAFs survived in an environment rich in SHH ligands. Flow cytometry was used to separate cells based on the expression of marker proteins including vimentin, α-SMA and cytokeratin 8. The CAFs were positive for α-SMA and vimentin and negative for cytokeratin 8, while the normal ovarian fibroblasts (NOFs) were positive for vimentin and negative for α-SMA and cytokeratin 8 (Figure 1A). The morphology of passage 3 CAFs and NOFs was mainly spindle-like intermixed with scalene triangular and polygonal cell morphologies (Figure 1B). To investigate whether CAFs can be activated by exogenous SHH ligands, the expression of PTCH in CAFs and NOFs was examined by immunofluorescence. CAFs and NOFs both expressed PTCH (P>0.05) (Figure 1C). However, after treatment with recombinant SHH (rSHH) at 1 μg/ml for 48 h, the expression of Gli-1 and PTCH in CAFs increased significantly, while the NOFs displayed no obvious difference (Figure 1D). Cyclopamine has anti-tumour properties via their direct binding to SMO. The expression of Gli-1 in CAFs was decreased in the presence of Cyclopamine (Figure 1E). Collectively, our observations suggest that the Hh signalling pathway can be activated by exogenous rSHH in CAFs but not in NOFs.

### SHH ligands promote the proliferation of CAFs *in vitro* by activating Hh signalling in CAFs

**Figure 2:**
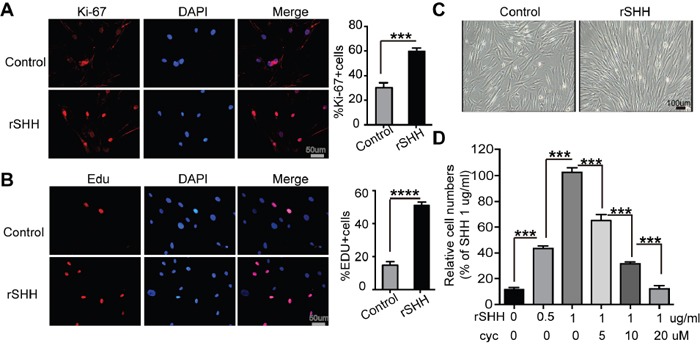
Exogenous SHH ligands promote the proliferation of CAFs *in vitro* **(A)** Representative fluorescent images and statistical analyses of the relative number of Ki-67-labeled (red) CAFs after 48 h treatment with rSHH (1 μg/ml). The nuclei were stained with DAPI (blue). **(B)** Representative fluorescent images and statistical analyses of the relative number of EDU-positive (red) CAFs after 48 h treatment with rSHH (1 μg/ml). The nuclei were stained with DAPI (blue). **(C)** Representative images of CAFs after 48 h treatment with rSHH (1 μg/ml). **(D)** Detection of relative viability of CAFs in the absence or presence of rSHH or Cyclopamine intervention at various doses for 48 h. Data are expressed as mean ± s.e.m. (***P<0.001, ****P<0.0001).

To determine the effect of SHH on the proliferation of CAFs *in vitro*, EDU assay and immunofluorescence for Ki-67 were performed in CAFs treated with rSHH for 48 h. The percentage of Ki-67- and EDU-positive CAFs in the rSHH-treated group was greater than that of the control group (Figure 2A and 2B). In addition, the CAFs multiplied rapidly after treatment with rSHH for 48 h (Figure 2C). Cell viability assays revealed that the proliferation of CAFs was notably enhanced after rSHH treatment in a dose-dependent manner (Figure 2D). In addition, the proliferation of CAFs was dramatically attenuated in the presence of Cyclopamine (Figure 2D). These results indicate that SHH ligand promoted the proliferation of CAFs *in vitro* by activating the Hh signalling pathway in CAFs.

### OC cell-derived SHH activates Hh signalling in CAFs

**Figure 3:**
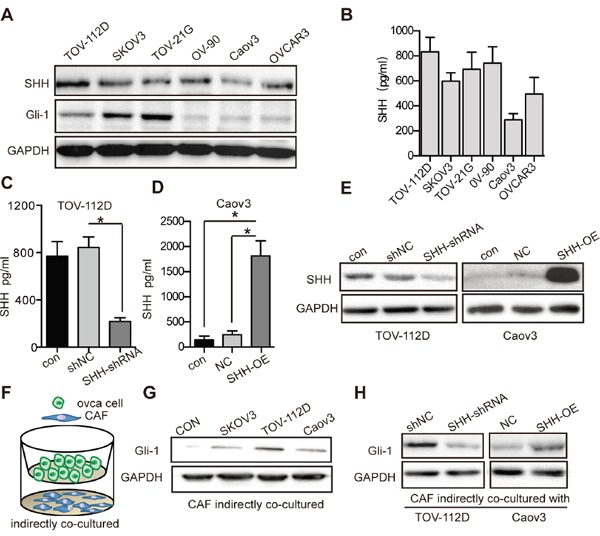
OC cell-derived SHH activates Hh signaling of CAFs **(A)** Western blot analysis of SHH and Gli-1 expression in OC cells. GAPDH served as the loading control. **(B)** ELISA of SHH ligand secreted by OC cells. **(C)** ELISA of SHH ligand secreted by TOV-112D cells stably transfected with either shNC or SHH-shRNA lentivirus. **(D)** ELISA of SHH ligand secreted by Caov3 cells stably transfected with either NC or SHH-OE lentivirus. **(E)** Western blot analysis of SHH and Gli-1 in TOV-112D and Caov3 cells stably transfected with lentivirus. **(F)** Schematic depicting of indirectly co-cultured OC cells and CAFs from the top to bottom. **(G)** Western blot analysis of Gli-1 expression in CAFs that were indirectly co-cultured with SKOV3, TOV-112D or CAOV3 cells. CAFs cultured alone were used as the control. **(H)** Western blot analysis of Gli-1 expression in CAFs that were indirectly co-cultured with TOV-112D and Caov3 cells stably transfected with lentivirus. Data are expressed as mean ± s.e.m. (*P < 0.05).

WB showed that Hh ligand expression and pathway activation occurred in OC cells (Figure 3A). To detect whether SHH ligands can be secreted to the extracellular space by OC cells, we used ELISA to determine the secretion of ligand into the media. The results showed that the level of SHH secreted by TOV-112D cells was relatively high, whereas that secreted by Caov3 cells was relatively low (Figure 3B). We also detected the invasive ability of these OC cells. TOV-112D cells possessed higher invasive ability, while Caov3 cells had lower invasive ability (Supplementary Figure 1D). Thus, TOV-112D and Caov3 cells were selected for transfection with SHH-knockdown or SHH-overexpressing lentivirus to alter SHH levels in OC. TOV-112D cells were lentivirally transfected with shRNA for knocking down SHH to establish TOV-112D SHH-shRNA cells and with control shRNA to establish TOV-112D shNC cells. The lentiviral vector encoding SHH gene was transfected into Caov3 cells to establish a stable SHH-overexpressing cell line called Caov3 SHH-OE, and the control lentiviral vector-transfected cell line was called Caov3 NC. WB and ELISA confirmed a significant decrease in the expression of SHH in the TOV-112D SHH-shRNA cells compared with that in the TOV-112D shNC cells (Figure 3C and 3E). The expression of SHH in Caov3 SHH-OE cells was prominently increased (Figure 3D and 3E). To determine whether OC cell-derived SHH can activate the Hh signalling pathway in CAFs, we developed an indirect co-culture model with OC cells seeded in the top chamber and CAFs seeded in the lower chamber for 2 days (Figure 3F). Compared with that in the CAFs cultured alone, the protein expression of Gli-1 in the CAFs co-cultured with TOV-112D, SKOV3 and Caov3 cells was increased in response to SHH secreted by OC cells. (Figure 3G). After co-culturing with TOV-112D SHH-shRNA cells, the expression level of Gli-1 was attenuated in the CAFs, while that in the CAFs co-cultured with Caov3 SHH-OE was dramatically increased (Figure 3H). These results suggest that OC cell-derived SHH activated the Hh signalling pathway in CAFs.

### Activation of the Hh signalling pathway in CAFs facilitates migration and capillary tube formation of LECs *in vitro*

**Figure 4:**
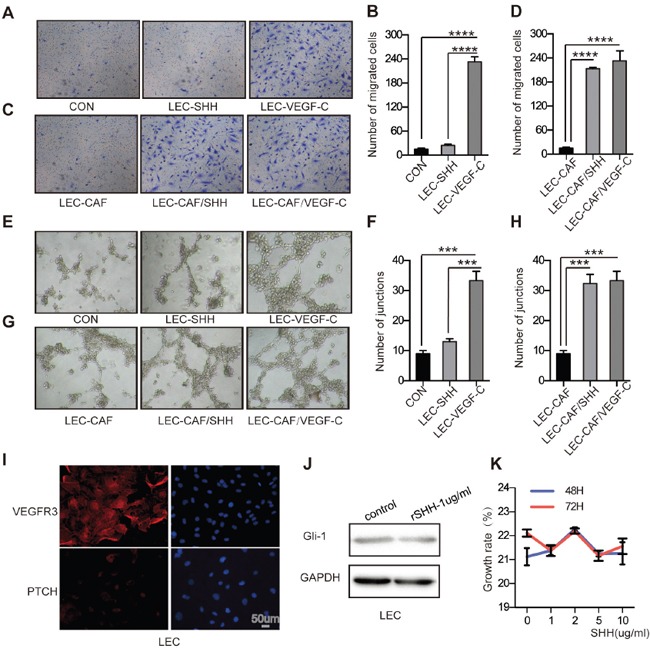
Activation of the Hh signaling pathway in CAFs promotes the migration and capillary tube formation of LECs *in vitro* **(A)** and **(B)** Representative images and statistical analyses of cellular migration of LECs treated with rSHH (1 μg/ml) or ECM. The group with VEGF-C (5 ng/ml) was the positive control. **(C)** and **(D)** Representative images and statistical analyses of migration of LECs in a co-culture invasion system with CAFs treated with or without rSHH. The group with VEGF-C (5 ng/ml) was the positive control. **(E)** and **(F)** Representative images and statistical analyses of *in vitro* capillary tube formation of LECs treated with rSHH (1 μg/ml) or ECM. LECs treated with VEGF-C (5 ng/ml) was the positive group. **(G)** and **(H)** Representative images and statistical analyses of capillary tube formation of LECs treated with supernatants from cultures of CAFs alone or from CAFs treated with rSHH (1 μg/ml). The group with VEGF-C (5 ng/ml) was the positive control. **(I)** Representative fluorescent images of VEGFR3- (red), PTCH- (red) and DAPI-labelled (blue) LECs. VEGFR3 is the positive marker for LECs as a membranous protein. **(J)** Western blot analysis of Gli-1 expression in LECs after 48 h treatment with rSHH (1 μg/ml). GAPDH served as the loading control. **(K)** CCK8 assay detected the growth rate of LECs at 48 h and 72 h after treatment with rSHH at various doses. The data are expressed as the mean ± s.e.m. (***P<0.001, ****P<0.0001).

Given the responsiveness of CAFs to Hh signalling, we wondered whether the activation of Hh signalling in CAFs was associated with elevated migration and capillary tube formation of LECs. The migration of LECs treated with rSHH was not significantly increased compared with no SHH treatment. (Figure 4A and 4B). Indirect co-culture of LECs with CAFs treated with rSHH increased the number of migratory LECs (P < 0.0001) compared with control LECs (Figure 4C and 4D). Model with VEGF-C(5ng/ml) was positive control. Tube formation assay showed rSHH did not directly accelerate the capillary tube formation in LECs (Figure 4E and 4F). However, tube formation of LECs incubated with supernatants from CAFs treated with rSHH was notably increased compared with that of LECs cultured with supernatants from untreated CAFs (Figure 4G and 4H). Model with VEGF-C was positive control. These results suggest that canonical Hh signalling in CAFs promoted the migration and capillary tube formation of LECs.

In addition, immunofluorescence analysis showed that LECs, which express VEGF-R3, expressed little if any PTCH (Figure 4I). After treatment with rSHH for up to 72 h, Gli-1 expression did not change in LECs (Figure 4J). In addition, we pre-treated LECs with rSHH (0–10 μg/mL, for 48 h or 72 h), but the growth rate of LECs was not different from that of the untreated control group (Figure 4K). The fact that LECs failed to respond to SHH suggests that endothelial cells are not directly responsive to canonical Hh signalling during Hh-mediated lymphangiogenesis.

### VEGF-C is a CAF-derived lymphangiogenesis factor induced by SHH

**Figure 5:**
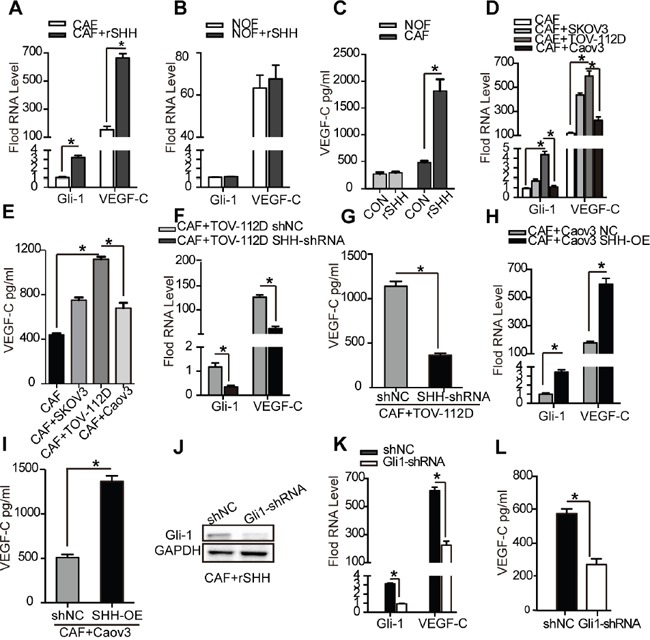
VEGF-C is a CAF-derived lymphangiogenesis factor induced by SHH **(A)** and **(B)** qRT-PCR analysis of Gli-1 and VEGF-C mRNA in CAFs and NOFs after 48 h treatment with rSHH (1 μg/ml). **(C)** ELISA of VEGF-C secreted by CAFs and NOFs after 48 h treatment with rSHH (1 μg/ml). **(D)** qRT-PCR analysis of Gli-1 and VEGF-C mRNA in CAFs and **(E)** ELISA of VEGF-C secreted by CAFs co-cultured with SKOV3, TOV-112D or Caov3 cells. **(F)** qRT-PCR analysis of Gli-1 and VEGF-C mRNA in CAFs and **(G)** ELISA of VEGF-C secreted by CAFs co-cultured with TOV-112D cells stably transfected with either shNC or SHH-shRNA lentivirus. **(H)** qRT-PCR analysis of Gli-1 and VEGF-C mRNA in CAFs and **(I)** ELISA of VEGF-C secreted by CAFs co-cultured with Caov3 cells stably transfected with either NC or SHH-OE lentivirus. **(J)** Western blot analysis of Gli-1 expression in CAFs stably transfected with NC or Gli-shRNA lentivirus in the presence of rSHH (1 μg/ml) for 48 h. **(K)** qRT-PCR analysis of Gli-1 and VEGF-C mRNA in CAFs and **(L)** ELISA of VEGF-C secreted by CAFs stably transfected with NC or Gli-shRNA lentivirus in the presence of rSHH (1 μg/ml) for 48 h. The data are expressed as the mean ± s.e.m. (*P<0.05).

To test whether lymphangiogenesis factors were secreted by fibroblasts, we allowed purified CAFs and NOFs to grow in the presence or absence of rSHH and derived conditioned media from them. Quantitative RT-PCR confirmed that the mRNA levels of Gli-1 and VEGF-C were increased (P < 0.05) in the CAFs treated with rSHH for 48 h (Figure 5A). However, the mRNA levels of Gli-1 and VEGF-C did not increase in the NOFs treated with rSHH for 48 h (Figure 5B). Meanwhile, ELISA of conditioned media showed an increased secretion of VEGF-C by the CAFs treated with rSHH (Figure 5C). To verify the effect of OC cell-derived SHH on the expression of VEGF-C in CAFs, CAFs were co-cultured with SKOV3, TOV-112D and Caov3 cells. Our results showed that OC cell-derived SHH increased expression of Gli-1 and VEGF-C in CAFs (Figure 5D and 5E). Furthermore, CAFs co-cultured with TOV-112D SHH-shRNA cells exhibited reduced Gli-1 and VEGF-C expression (Figure 5F and 5G). In addition, after the CAFs were co-cultured with Caov3 SHH-OE cells, there was a notable increase in the expression of Gli-1 and VEGF-C (Figure 5H and 5I). Thus, there was a positive correlation between Gli-1 and VEGF-C expression in CAFs with SHH expression in OCs. To further detect whether VEGF-C is a Hh-responsive gene in CAFs, lentiviral shRNA for knocking down Gli-1 or control shRNA were transfected into CAFs to generate CAFs Gli1-shRNA or CAFs shNC, respectively. (Figure 5J). The mRNA levels of Gli-1 and VEGF-C were dramatically decreased in the CAFs Gli1-shRNA treated with rSHH (P < 0.01) in comparison to those in the CAFs shNC (Figure 5K). Additionally, ELISA showed that the secretion of VEGF-C was attenuated in the CAFs Gli1-shRNA treated with rSHH (Figure 5L). Together, these results demonstrate that Hh induced CAFs to secrete the lymphangiogenesis factor VEGF-C.

### Paracrine signalling of tumour-initiated SHH in CAFs accelerates lymphangiogenesis *in vivo*

**Figure 6:**
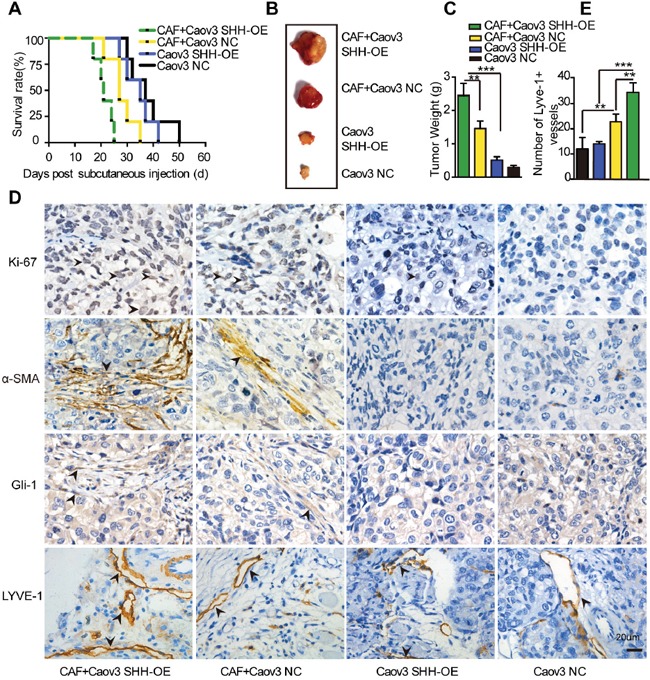
Paracrine signaling of tumour-derived SHH in CAFs promotes lymphangiogenesis *in vivo* **(A)** Growth properties of subcutaneous tumours in mice derived from Caov3 SHH-OE or Caov3 NC cells alone or co-injected with CAFs. Kaplan-Meier plot of time to tumour progression, as evidenced by survival rates over time (n=5). **(B)** Bright field images of subcutaneous tumours from each group (n=5) 20 days after tumour implantation. **(C)** Graphical representation of the weights of subcutaneous tumours from each group 20 days after tumour implantation. **(D)** Immunohistochemical staining for Ki-67, α-SMA, Gli-1 and LYVE1 in tumour sections from mice in each group. **(E)** Graphical representation of the number of LYVE1+ vessels counted from IHC images in panel D. (*P<0.05, **P<0.01, ***P<0.001).

The observation that SHH secreted by Caov3 SHH-OE cells promoted VEGF-C expression in CAFs by activating the Hh pathway led us to hypothesize that the paracrine signalling of SHH in fibroblasts was associated with tumour growth and lymphangiogenesis *in vivo*. We subcutaneously co-injected CAFs and Caov3 SHH-OE or Caov3 NC cells into Balb/c-null mice. Co-injection of CAFs and Caov3 SHH-OE cells significantly accelerated tumour progression and resulted in larger tumours compared to the injection of Caov3 SHH-OE (P < 0.01) or Caov3 NC cells (P < 0.05) alone, or the co-injection of CAFs with Caov3 NC cells (Figure 6B and 6C). The survival rate of mice with tumours derived from Caov3 SHH-OE cells co-injected with CAFs was reduced compared to that of mice with tumours derived from Caov3 NC cells co-injected with CAFs (P < 0.05) or from Caov3 SHH-OE or Caov3 NC cells alone (Figure 6A). These findings indicate that paracrine signalling of OC cell-derived SHH in CAFs facilitated tumour growth.

IHC analysis of tumour sections confirmed the elevation of the proliferation marker Ki-67 and stromal Gli-1 expression, as well as the increase in α-SMA staining and resultant expression of the lymphatic marker LYVE1, in the tumours from mice co-injected with Caov3 SHH-OE cells and CAFs compared with that in the tumours from mice in co-injected with Caov3 NC cells and CAFs (Figure 6D). Furthermore, IHC analysis identified that the tumours from injection of Caov3 SHH-OE cells alone and Caov3 NC cells alone barely expressed Ki-67, stromal Gli-1 and α-SMA. The number of LYVE1+ vessels was attenuated in these groups compared with the group with co-injection of CAFs (Figure 6E). Collectively, our results demonstrate that paracrine Hh signalling in CAFs promoted tumour growth and enhanced lymphangiogenesis.

### Inhibition of Hh signalling in CAFs blocks its effect on lymphangiogenesis *in vitro* and *in vivo*

**Figure 7:**
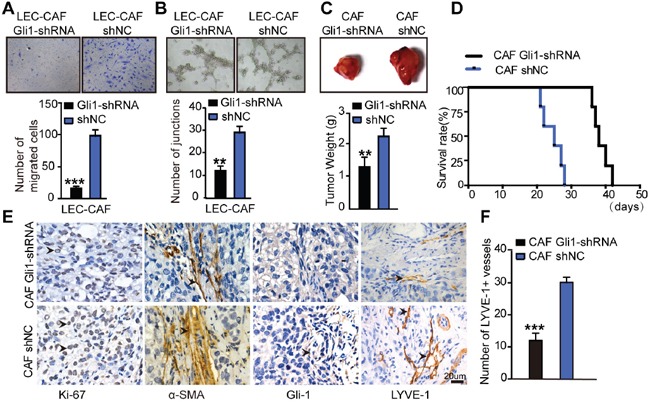
Inhibition of Hh signaling in CAFs blocks its effects on lymphangiogenesis *in vivo* and *in vitro* **(A)** Representative images and statistical analyses of migration of LECs in a co-culture invasion system with CAFs stably transfected with shNC or Gli-shRNA lentivirus in the presence of rSHH (1 μg/ml). **(B)** Representative images and statistical analyses of capillary tube formation of LECs treated with supernatants from cultures of CAFs stably transfected with shNC or Gli-shRNA lentivirus in the presence of rSHH (1 μg/ml). **(C)** Bright field images and graphical representation of the weights of tumours of subcutaneous tumours from mice (n=5) bearing Caov3 SHH-OE cells co-injected with CAFs stably transfected with shNC or Gli-shRNA lentivirus 20 days after tumour implantation. **(D)** Growth properties of subcutaneous tumours in mice derived from Caov3 SHH-OE cells co-injected with CAFs stably transfected with shNC or Gli-shRNA lentivirus. Kaplan-Meier plot of time to tumour progression, as evidenced by survival rates over time. **(E)** IHC staining for Ki-67, α-SMA, Gli-1 and LYVE1 in tumour sections from mice in the two groups. **(F)** Graphical representation of the number of LYVE1+ vessels counted from IHC images in panel E. (**P<0.01, ***P<0.001).

Migration assay confirmed that treatment of rSHH in CAFs Gli1-shRNA attenuated the number of migrating LECs (P < 0.0001) compared with that with the CAFs shNC (Figure 7A). In addition, we observed a decreased capillary tube formation in LECs incubated with supernatants from CAFs Gli1-shRNA treated with rSHH (P < 0.001) compared with that in LECs grown with supernatants derived from CAFs shNC (Figure 7B). We co-injected Caov3 SHH-OE cells with CAFs Gli1-shRNA or CAFs shNC in xenograft experiments. Tumours arising from the co-injection of Caov3 SHH-OE cells with CAFs Gli1-shRNA were smaller than those arising from the co-injection of CAFs shNC (Figure 7C). A significant longer survival period was also observed for mice with tumours derived from Caov3 SHH-OE cells co-injected with CAFs Gli1-shRNA compared to that of mice with tumours derived from Caov3 SHH-OE cells co-injected with CAFs shNC (Figure 7D). Subsequently, IHC analysis of tumour sections showed reduced staining of the proliferation marker Ki-67 and attenuated α-SMA staining, as well as decrease in the resultant expression of the lymphatic marker LYVE1, in the group co-injected with CAFs Gli1-shRNA compared with that of the group co-injected with CAFs shNC. In addition, the stroma in the group co-injected with CAFs Gli1-shRNA barely expressed Gli-1 (Figure 7E). The number of LYVE1+ vessels in the group co-injected with CAFs Gli1-shRNA was reduced compared with that in co-injected with CAFs shNC (Figure 7F). Taken together, our data shows here that the effect of CAFs on tumourigenesis and lymphangiogenesis in OC depended on OC cell-derived SHH and Hh signalling activity in CAFs.

## DISCUSSION

**Figure 8:**
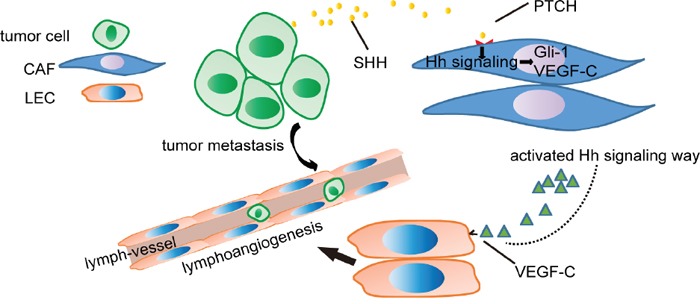
Schematic of OC cell-mediated Hh signalling in CAFs and the VEGF-C feed-forward loop Hh ligands secreted by OC cells activate Hh signalling in CAFs. Then, Hh signalling in CAFs results in the expression of VEGF-C that facilitates the migration and capillary tube formation of LECs, thus promoting tumour metastasis.

The key finding from this study is that CAFs promote lymphangiogenesis in OC via the Hh/VEGF-C signalling axis (Figure 8). We demonstrated here that OC cell-derived SHH activated Hh signalling in CAFs and promoted the proliferation of CAFs. Activation of the Hh signalling pathway in CAFs facilitated the migration and capillary tube formation in LECs. VEGF-C was identified as a candidate lymphangiogenesis factor secreted by CAFs in response to Hh stimulation. Blocking of Hh signalling in CAFs decreased VEGF-C expression and diminished the positive role of CAFs in supporting tumourigenesis and lymphangiogenesis. Our findings demonstrated the pivotal role of Hh signalling in the tumour stroma as a regulator of lymphangiogenesis. CAFs constitute a supportive niche for cancer lymphangiogenesis via Hh/VEGF-C signalling, and targeting this signalling may be a new therapeutic strategy for OC.

Previous studies have indicated that tumour cells synthesize and respond to the Hh ligand in an autocrine-juxtacrine manner [17, 18]. However, our data suggested a paracrine mode of the canonical Hh signalling pathway in OC. Our data are consistent with previous findings that SHH is overexpressed in pancreatic tumour cells and that tumour cell-derived SHH activates the Hh signalling pathway in pancreatic stellate cells instead of in tumour cells [19]. Our study provides evidence that Hh signalling is an important link between OC cells and stromal cells. We demonstrated that the CAFs were responsive to Hh and that SHH originating from the epithelium activated canonical Hh signalling in the adjacent mesenchyme. Our study of Hh signalling between the two cell types sheds new light on the understanding of lymphangiogenesis in OC and enables the identification of a potential therapeutic target.

The importance of paracrine Hh signaling lies in the nurture and maintenance of tumor microenvironment [20, 21]. An increasing amount of evidence has demonstrated the importance of the involvement of stromal components, which account for 7%–83% of the tumour tissue, in the pathogenesis and progression of OC [22, 23]. In this study, our data showed that CAFs were responsive to Hh signalling and OC cell-derived SHH signalling to CAFs promoted the production of the lymphangiogenesis factor VEGF-C. Inhibition of Hh signalling in CAFs blocked their pro-lymphangiogenic effects. Similarly, it has been reported that OC cell-derived SHH induced human ovarian carcinoma-associated mesenchymal stem cells to express BMP4, which promoted chemotherapy resistance in OC [24]. Taken together, our findings further strengthen the idea that tumour-stromal interactions are critical for tumour growth and progression. Furthermore, the interactions of tumour-associated macrophages and SHH-positive medulloblastoma cells have been reported to contribute to tumour growth [25]. Consistent with these observations, our study revealed an effect of Hh signalling on the OC microenvironment and demonstrated that Hh signalling was amplified by the crosstalk between the tumour and stroma, resulting in promoting tumour metastasis. However, the most current therapeutic agents targeting OC overlook the importance of the tumour-supportive microenvironment [26]. Taken together, these data suggests that the inhibition of the stromal Hh response instead of directly targeting the tumour cells might be a potential therapeutic approach to treat OC metastasis.

Studies have demonstrated that VEGF-C is overexpressed in many cancers and can promote the formation of lymphatic vessels and metastasis of tumour cells to lymph nodes via the activation of VEGF receptor-3 [27]. The incidence of lymph node metastasis in clinically advanced EOC is considerable, and lymphatic vessels have been reported as an important route contributing to the metastasis of solid tumours [16]. Previous studies have also illustrated that VEGF-C is a potential clinical marker in breast cancer or OC patients and compensates for the shortage of CA125 [28]. Here, we demonstrated that the CAFs upregulated VEGF-C in response to Hh signalling and promoted the migration and capillary tube formation of LECs. The level of VEGF-C is related to the microlymphatic vessel density. Our data showed that OC cell-derived Hh signalling of CAFs resulted in a significant increase in the number of LYVE1+ vessels *in vivo*. Our study is the first to suggest that CAFs are a source of VEGF-C in the tumour microenvironment. Similarly, tumour-associated macrophages were also found to produce VEGF-C in the tumour microenvironment [29]. Elucidation of the mechanisms that regulate VEGF-C might provide new insights for cancer therapies. Therefore, it is imperative to evaluate stroma-targeted therapeutics in the presence of tumour stroma in humans. The expression of VEGF-C in different tumour types may be regulated by various signalling factors including cytokines, hormones, hypoxia, etc. [30]. Thus, Hh-driven VEGF-C secretion by CAFs partly contributes to lymphangiogenesis. Our data supplemented these findings.

In conclusion, the identification that tumour-derived SHH can induce stromal VEGF-C to promote lymphangiogenesis further supports the importance of the tumour microenvironment. It is important to evaluate stroma-targeted therapeutics in the presence of tumour stroma in humans. Our findings have helped to elucidate the mechanism of the pro-tumourigenic functions of CAFs. Finally, these results provide evidence to support the clinical application of Hh inhibitors in OC.

## MATERIALS AND METHODS

### Cell culture

LECs were purchased from ScienCell (San Diego, California, USA)and were grown in Endothelial Cell Medium (ECM) according to the manufacturer's instruction. Human EOC cell lines SKOV3, TOV-112D, TOV-21G, A2780, OV-90, OVCAR3 and Caov3 were purchased from American Type Culture Collection (Rockville, MD, USA). SKOV3 cells were cultured in McCoy's 5A medium supplemented with 10% foetal bovine serum (FBS). OVCAR3 cells were cultured in DMEM supplemented with 10% FBS and 0.01 mg/ml bovine insulin. Caov3 cells were cultured with DMEM containing 10% FBS. TOV-112D, TOV-21G and OV-90 cells were cultured in a 1:1 mixture of medium 199 (Invitrogen, Carlsbad, CA) and medium 105 (Sigma-Aldrich, St Louis, MO) with 10% FBS. NOFs were obtained from normal ovarian tissues, and CAFs were obtained from ovarian tumour tissues following procedures as previously described [31]. All these cell lines were grown in a humidified atmosphere with 5% CO_2_ at 37°C.

### OC samples

Human OC tissue specimens used for the isolation of CAFs and NOFs were obtained from surgically removed tissues of patients in Wuhan TongJi Hospital of Huazhong University of Science and Technology (Wuhan, China); the patients had not received any preoperative radiotherapy or chemotherapy. Among these patients, 12 were diagnosed with ovarian serous cystadenocarcinoma, 3 with ovarian mucinous cystadenocarcinoma, 3 with ovarian clear cell carcinoma and 2 with endometrioid adenocarcinoma. All of the tumour samples were obtained from primary tumour sites. The mean age of the patients was 49.6 (range 30–70) years. Normal ovarian tissues were obtained from noncancerous prophylactic oophorectomy specimens. Within the 20 epithelial ovarian carcinoma cases, 4 cases were in stage I, 6 in stage II, 5 in stage III and 5 in stage IV, based on the staging system of the International Federation of Gynaecology and Obstetrics (FIGO stage). The CAFs used in the study were obtained from 12 ovarian serous cystadenocarcinoma samples.

### Reverse transcription quantitative real-time PCR (RT-qPCR)

Total RNA from cells was extracted using TRIzol reagent (Invitrogen, Carlsbad, CA, USA) and reverse transcribed into cDNA using M-MLV reverse transcriptase (Takara, Japan). RT-PCR was performed by using the Bio-Rad CFX96 system with SYBR Green. The sequences of the primers used are as follows: Gli1, 5’-TCCTACCAGAGTCCCAAGTT-3’ (forward) and 5’-CCCTATGTGAAGCCCTATTT-3’ (reverse); PITH, 5’-CCACGACAAAGCCGACTACAT-3’ (forward) and 5’-GCTGCAGATGGTCCTTACTTTTTC-3’ (reverse); SMO,5’-CCTTTGGCTTTGTGCTCATTACCTT-3’ (forward) and 5’-CGTCACTCTGCCCAGTCAACCT-3’ (reverse); VEGF-C, 5’-AACCATGAACTTTCTGCTGTC TTG-3’ (forward) and 5’-TTCACCACTTCGTGATGA TTCTG-3’ (reverse); 18S, 5’-CGTCTGCCCTATCAA-3’ (forward) and 5’-ATGTGGTAGCCGTTT-3’ (reverse). The amplification protocols were as follows: 95°C for 60 sec and 40 cycles at 95°C for 15 sec, 57°C for 15 sec and 72°C for 45 sec.

### Indirect co-culture of CAFs/OC cells

OC cells were co-cultured with CAFs using transwells (6-well plate with 3 μm inserts) (Millipore German). OC cells (2×10^5^ cells) were seeded onto the top chamber in 1.5 ml media, and CAFs (5×10^5^ cells) were seeded into the lower chamber in 2 ml media for 2 days.

### Preparation of conditioned media

CAFs, digested with 0.125% trypsin–EDTA, were seeded in six-well plates at 2×10^6^ cells/ml and then incubated at 37°C with 5% CO_2_ for 2 h. At the end of the incubation time, the supernatants were aspirated and replaced by ECM in the presence or absence of rSHH (1 μg/ml). After 12 h, the conditioned media were collected and centrifuged to remove cellular debris, and the supernatants were stored at 4°C. The conditioned media were used without dilution.

### Western blot analysis

Total cell lysates were prepared in RIPA lysis buffer (Beyotime, Shanghai, China) supplemented with a protease inhibitor cocktail (Roche). Protein concentrations were determined using the bicinchoninic acid (BCA) assay (Thermo Scientific), and 40 ng of total lysate for each sample was subjected to SDS-PAGE followed by blotting with the indicated primary antibodies. The antibody against Gli1 (#3538) was purchased from Cell Signalling Technology (Beverly, MA, USA). Antibodies against PTCH (sc-6147) and SHH (sc-1194) were obtained from Santa Cruz Biotechnology (Dallas, Texas, USA). The antibody against GAPDH (ab9485) was obtained from Abcam Biotechnology (Abcam, CA, USA). The membranes were incubated with primary antibodies at 4°C overnight. After washing with three times with TBST, the membranes were incubated with the corresponding HRP-linked secondary antibodies (Santa Cruz Biotechnology), and the signals were detected by the enhanced ECL system (Pierce).

### Immunofluorescence staining

Cells were subjected to immunofluorescence staining on coverslips. After fixation, the cells were incubated with primary antibodies against α-SMA (ab7817, Abcam), PTCH and Ki-67 (ab15580, Abcam) and then incubated with FITC-conjugated secondary antibody or Alexa Fluor 594-conjugated secondary antibody (Invitrogen). The coverslips were counterstained with 40,6-diamidino-2-phenylindole (DAPI) and imaged using an Olympus BX53 microscope (Olympus, Tokyo, Japan).

### Transfection of lentivirus

Lentiviral delivered shRNA was used to knock down SHH in TOV-112D cells to generate the TOV-112D SHH-shRNA cells, or blank plasmid was used to generate the TOV-112D shNC cells. Lentiviral overexpressing SHH gene was used to transfect Caov3 cells to generate the Caov3 SHH-OE cells, or blank plasmid was used to generate the Caov3 NC cells. Lentiviral delivered shRNA was used to knock down Gli-1 in CAFs to generate the CAFs Gli1-shRNA, or blank plasmid was used to generate the CAFs shNC. GFP-based flow sorting was performed to select the cells that were stably transduced with lentiviruses. All lentiviruses were obtained from GeneChem (Shanghai, China).

### Animal assay

The animal experiments were performed with approval of the Committee on Ethics of Animal Experiments in the Hubei province. Four- to six-week-old female Balb/c-null mice were housed and maintained in laminar flow cabinets under specific pathogen-free conditions. Cells (3×10^6^) alone or in combination with CAFs (1×10^4^) were subcutaneously injected into the right back of mice. The animals were monitored every 2 days. Animal experiments to measure survival rates and for comparing tumour growth were performed independently. The group of animals used for the detection of survival rates was monitored until all mice spontaneously died. The other group of animals was used for the measurement of tumours. Approximately 20 days later, all animals were euthanized, and the xenografts in each group were collected for further immunohistochemical studies.

### ELISA

This assay was conducted using VEGF-C or SHH ELISA kits according to the manufacturer's instructions (RayBiotech, Atlanta, Georgia USA). The supernatants were subjected to ELISA in triplicate. Absorbance at 540 nm was determined using an automated microplate reader (SpectraMax 190, Molecular Devices, USA). VEGF-C or SHH concentrations were computed with reference to standard curves derived from purified VEGF-C or SHH supplied with the respective ELISA kits.

### Transwell migration and tube formation assays

For migration assays, LECs were seeded in transwell culture dishes (8 μm pores, 1.0 × 10^4^ cells/well) coated with fibronectin (2 mg/cm^2^, Millipore) in 100 μl of ECM containing 5% foetal calf serum. Five hundred microlitres of ECM containing 5.0×10^5^ CAFs or 5.0×10^5^ CAFs treated with rSHH (1 μg/ml) were added to the lower chambers. rSHH (1 μg/ml) was added to the upper chambers; ECM supplemented with rVEGF-C (5 ng/ml) served as the positive control, and ECM alone served as the negative control. After 24 h, the migrated cells were fixed for 20 min with 4% paraformaldehyde, stained with 0.05% crystal violet for 10 min, photographed and counted. Tube formation on Matrigel was carried out as follows. Briefly, LECs were seeded in Matrigel-coated 96-well tissue culture plates (1.0×10^4^ cells/well) and incubated with 1 μg/ml rSHH or conditioned media from CAFs. ECM supplemented with rVEGF-C (5 ng/ml) served as the positive control, and ECM alone served as the negative control. Phase-contrast images of tubes were taken after 3, 6, 8 and 12 h at 10× magnification, and total tube length and total number of branch points were quantified in triplicate wells by an Olympus IX73 microscope (Olympus, Tokyo, Japan).

### Immunohistochemistry (IHC)

Paraffin-embedded tissue sections were first deparaffinized. After antigen retrieval, the slides were incubated with 3% H_2_O_2_ to inhibit endogenous peroxidase, blocked with bovine serum for 30 min and incubated with anti-SHH (ab53281, abcam), anti-αSMA, anti-Gli1(NB600-600, Novus), anti-LYVE1 (ab14917, Abcam) and anti-Ki67 antibodies overnight at 4°C. The next day, the slides were exposed to horseradish peroxide-conjugated secondary antibodies for 30 min. Finally, the slides were developed in DAB solution for optimal staining intensity. The intensity of staining was subjectively graded on a four-point scale with 0 indicating the absence of staining, 1 indicating the lowest level detectable and/or non-homogeneous weak staining, 2 indicating moderate homogeneous staining and 3 indicating intense homogeneous staining. The percentage of positive tumour cells were scored as follows: 1, no detectable immunostaining; 2, focal, <25% of cells immunostained; 3, regional, between 25% and 50% of cells immunostained; 4, diffuse, >50% of cells immunostained. The immunostaining intensity was evaluated by two independent observers who were blind to the clinical data. IHC for LYVE1 was used to detect lymphatic vessels. The number of vessels was counted in high magnification fields (400×) for each group (n ≥3). At least five microscopic fields per mouse were scored for each genotype.

### Statistics

The data are presented as the mean value ± s.e.m from at least three independent experiments. Statistical analyses were performed with Prism 6.0 GraphPad software. Single comparisons between two groups were done by Student's t-test. Comparisons between multiple groups were done by one-way ANOVA followed by Tukey's post-test. P values < 0.05 were considered significant.

## SUPPLEMENTARY MATERIALS FIGURE


