# Supplementary material for: Human CAFs promote lymphangiogenesis in ovarian cancer via the Hh-VEGF-C signaling axis
Source: Oncotarget. 2017 Jun 27;8(40):67315–28. doi: 10.18632/oncotarget.18621 (PMC5620175; doi:10.18632/oncotarget.18621)
Supplement: Supplementary file 1 [file oncotarget-08-67315-s001.pdf]

Human CAFs promote lymphangiogenesis in ovarian cancer via the Hh-VEGF-C signaling axis

SUPPLEMENTARY MATERIALS

SUPPLEMENTARY FIGURE

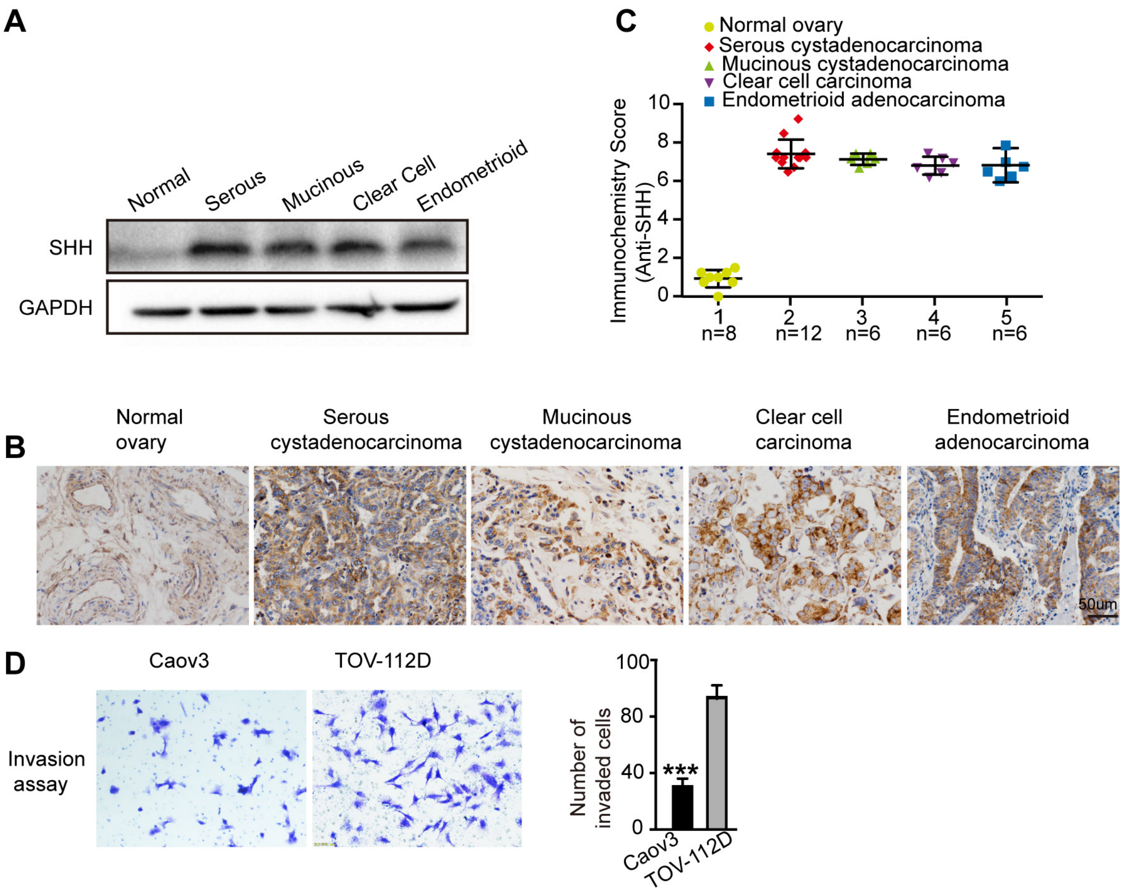

Supplementary Figure 1: (A) Western blot analysis of SHH expression in the four different types of OC tissues and normal ovarian tissues. GAPDH served as the loading control. **(B)** Immunohistochemistry (IHC) detection of SHH in the four different types of OC tissues and normal ovarian tissues. **(C)** Scoring of SHH in the four different types of OC tissues and normal ovarian tissues of the above IHC stained tissues. **(D)** Representative images and statistical analysis of cellular invasion of Caov3 and TOV-112D cells. The data are expressed as the mean ± s.e.m. (***P<0.001).
